# Identifying the barriers to conducting outcomes research in integrative health care clinic settings - a qualitative study

**DOI:** 10.1186/1472-6963-10-14

**Published:** 2010-01-14

**Authors:** Marja J Verhoef, Andrea Mulkins, Ania Kania, Barbara Findlay-Reece, Silvano Mior

**Affiliations:** 1 Department of Community Health Sciences, University of Calgary, Calgary, Canada; 2 Samueli Institute, Alexandria, Virginia USA; 3 Omni Health, White Rock, Canada; 4 Department of Research, Canadian Memorial Chiropractic College, Toronto, Canada

## Abstract

**Background:**

Integrative health care (IHC) is an interdisciplinary blending of conventional medicine and complementary and alternative medicine (CAM) with the purpose of enhancing patients' health. In 2006, we designed a study to assess outcomes that are relevant to people using such care. However, we faced major challenges in conducting this study and hypothesized that this might be due to the lack of a research climate in these clinics. To investigate these challenges, we initiated a further study in 2008, to explore the reasons why IHC clinics are not conducting outcomes research and to identify strategies for conducting successful in-house outcomes research programs. The results of the latter study are reported here.

**Methods:**

A total of 25 qualitative interviews were conducted with key participants from 19 IHC clinics across Canada. Basic content analysis was used to identify key themes from the transcribed interviews.

**Results:**

Barriers identified by participants fell into four categories: organizational culture, organizational resources, organizational environment and logistical challenges. Cultural challenges relate to the philosophy of IHC, organizational leadership and practitioner attitudes and beliefs. Participants also identified significant issues relating to their organization's lack of resources such as funding, compensation, infrastructure and partnerships/linkages. Environmental challenges such as the nature of a clinic's patient population and logistical issues such as the actual implementation of a research program and the applicability of research data also posed challenges to the conduct of research. Embedded research leadership, integration of personal and professional values about research, alignment of research activities and clinical workflow processes are some of the factors identified by participants that support IHC clinics' ability to conduct outcomes research.

**Conclusions:**

Assessing and enhancing the broader evaluation culture of IHC clinics prior to implementing outcomes research may be a critical step towards ensuring productive and cost-effective research programs. However, as IHC clinics are often complex systems, a whole systems approach to research should be used taking into account the multidimensional and complex nature of such treatment systems so that the results are useful and reflect real life.

## Background

Integrative health care (IHC) is an interdisciplinary blending of conventional medicine and complementary and alternative medicine (CAM) for the purpose of enhancing patients' health [1]. The blending of these approaches ranges from "combining treatments from conventional medicine and complementary/alternative therapies for which there is some high-quality evidence of safety and effectiveness" [2] to "the practice of medicine that reaffirms the importance of the relationship between practitioner and patient, focuses on the whole person, is informed by evidence, and makes use of all appropriate therapeutic approaches, healthcare professionals and disciplines to achieve optimal health and healing" [3]. The increasing development of and demand for IHC clinics to provide a comprehensive evidence-informed approach to health and healing has prompted the need for regular assessment of patient-centered outcomes to assess the value of such models. Despite this realization, few CAM/IHC clinics in North America have achieved robust and productive outcomes research programs.

Based on our team's experiences working in IHC clinics [4,5], outcomes assessment is challenging, especially finding and choosing outcome measures that are appropriate and relevant to practitioners, patients, and administrators, and that do not burden the patient or practitioner. In addition, many of the outcome measures used to date tend to reflect a biomedical paradigm and measure disease oriented clinical and health status outcomes, rather than outcomes that capture the patients' lived experiences and self-identified health goals. This may, partly, be attributed to the need to demonstrate 'credible' results to the biomedical community.

In addition to physical health and well-being, the goals of IHC include outcomes such as self-awareness, transformation, motivation, balance, feeling connected and patient empowerment [6]. However, the measurement and evaluation of these outcomes in IHC remains rare. For example, a review of evaluations of primary and community care services that include CAM in the UK, found that of the 25 services identified, the most commonly used outcome measures were data extracted from referral forms, service billings, patient satisfaction surveys, and patient health status questionnaires [7]. Outcomes specific to CAM and IHC, as identified above, were often not assessed at all. A well-known instrument such as the MYMOP [8], an individualized assessment of outcomes that are most important to individual patients, was used in only 4 of the 25 service evaluations. IHC evaluations in North America also included only a limited range of outcome measures, and most of these were not CAM and IHC specific [9-13]. One study assessing patient outcomes in a collaborative model involving chiropractors and physicians did include the MYMOP along with pain and quality of life measures [14]. A recent study that evaluated an IHC model at the University of Michigan was one of a few that used a relevant but not yet validated outcome measure (the Holistic Health Questionnaire) that addresses the various components of health identified within an IHC philosophy [15].

Based on the literature and our previous work we developed a 'package of outcome measures' (Additional file 1) in 2006 to enable IHC clinics to better understand if their unique approaches foster and enhance healing and to facilitate the comparison of different integrative models. This package consisted of instruments to assess concepts that were identified as central to IHC, closely capture the patients' lived experiences with IHC, and align clinical measures and research instruments with the philosophies and broad goals of IHC clinics. This package was piloted at four IHC clinics in Canada and the US. The purpose of the pilot study was to: 1) explore the feasibility of systematic outcomes data collection in IHC clinics, and 2) to assess which combination of outcome measures best captures healing experiences of patients in order to evaluate IHC clinics.

Despite strong study support from clinic leadership, implementation and recruitment for this pilot project was challenging. Although a significant amount of time was spent with each clinic to ensure that study protocols were modified and tailored to each participating clinics' critical paths and intake processes, it never became fully integrated into the daily practices and activities of the clinic. Challenges with patient recruitment were related to which patients should be approached for the study, when they should be approached, and by whom. Retention of participants was also a problem as many participants were lost to follow-up or withdrew from the study. The main reasons cited for withdrawal included health issues, no longer attending the clinic, and lack of time. Administration of the outcomes package at baseline was also problematic as it increased the large amount of paper work that new patients were required to complete. From our observations and interviews it also appeared that the clinics were not just struggling with the research process, but were subtly resistant to the notion of conducting research within their clinic, as such an evaluation process could be construed as interfering with their carefully conceived patient services. Finally, it appeared that participants saw no benefit from a research-in-practice cycle where research informs and enhances clinical practice.

In addition to the logistical issues, we learned of some major shortcomings of the outcomes package. Through participant feedback, it became apparent that the package did not focus enough on the positive aspects of well being and that it centered too much on ill-health. Similarly, several participants expressed that the package was more appropriate for individuals who were suffering from serious and chronic health concerns and suggested that most of the questions were not applicable for those who are attending clinics for health promotion and/or prevention reasons. This feedback brings forth the recognition that outcomes used to evaluate IHC not only need to be in line with the IHC philosophy of care but should also consider why people seek out IHC.

Since these issues were not exclusive to one or two clinics, we realized that successful outcomes research in IHC clinic settings is much more than having effective recruitment strategies, feasible data collection processes, and relevant patient-friendly outcome measures. We hypothesized that factors affecting a clinic's ability to successfully conduct outcomes research may arise out of lack of a culture in which research is considered important. We refer to a 'research culture' as a shared system of values, beliefs and attitudes within an organization which shapes and influences how employees engage in research activities. Research culture also describes the degree to which an organization defines itself in relation to research and how integral it is to practice [16]. A lack of research culture is not an issue that is limited to IHC, as it has also affected such fields as primary care, counseling, education and kinesiology [17-21].

To facilitate the development of evidence-informed IHC practices, we developed a second phase to the original study to further explore the issues related to research culture. The objectives of this study were to: 1) understand and identify the reasons why IHC clinics are not conducting outcomes research, and 2) to determine the factors necessary for research programs to be successful and sustainable.

## Methods

Due to the limited amount of information in the IHC field and the exploratory nature of the study objectives, a qualitative study design was used. Forty IHC clinics were identified across Canada through a combination of internet-based searches, informal networking among Canadian clinics, and a list of IHC clinics identified by Gaboury [22]. Clinics were selected based on the following inclusion criteria: 1) multi-disciplinary approach, 2) a written mission statement outlining its role in healing and curing, 3) There are at least two CAM practitioners who practice different healing modalities, and 4) If there is not a medical doctor who practices on site, there must be some kind of formal communication strategy that outlines some level of collaboration between the clinic and physicians in the community. Letters of invitation were mailed to 80 individuals at 40 clinics. Approximately one to two individuals were contacted at each clinic, holding prominent roles, such as Clinic Director, Clinic Manager, Medical Director, Patient Care Coordinator and practitioners (physicians and CAM practitioners including naturopathic doctors, Traditional Chinese Medicine practitioners, and chiropractors).

Each study participant took part in an in-depth, semi-structured telephone interview, approximately 0.5-1 hour in length. They were conducted by two trained interviewers, based in British Columbia. The interview guide is included in Additional file 2. Because the concept of 'research culture' is somewhat abstract, this term was used interchangeably with the words 'climate' and 'environment' in the interviews. Nondirective prompts were used to encourage discussion of important issues that arose. Participants were also asked about their role in the clinic, time in the position and past research training.

Interviews were audio-taped and transcribed verbatim for subsequent analysis. Data collection and analysis were iterative, meaning that each transcript was analyzed before a subsequent interview took place. This process helped to assess the point where data saturation was reached. A basic content analysis approach was used, which involved a systematic process of manually indexing, coding, categorizing and interpreting [23]. Transcripts were first analyzed line by line to identify concepts and then themes and categories were developed into a coding framework for application to the data. During this process, new themes developed and others changed. Analysis was independently conducted by two of the researchers. Themes emerging from the independent analyses were compared and discussed until consensus was reached.

Ethics approval was received from the Office of Medical Bioethics in the Faculty of Medicine at the University of Calgary (reference number 20222) on July 3, 2009 as a modification of the ethics approval received on May 30, 2006 for the initial pilot study.

All individuals voluntarily participated in the interviews. Prior to the interview, all participants were informed of: 1) the purpose and objectives of the study, 2) their rights to decline participation at any point during the interview or thereafter, and 3) how confidentiality of their persons and respective clinics would be protected. Any questions from the participants were addressed and informed consent was obtained verbally over the telephone by the interviewer prior to each telephone interview.

## Results

A brief description of the participating clinics can be found in Table 1. 19/40 clinics agreed to participate in the study with 25/80 individuals initially approached agreeing to an interview. In most clinics one person was designated to be interviewed, in six others, two or three people volunteered. Reasons for the low response included lack of time, not feeling as though they had anything to contribute, being a newly established clinic, working in a new position and lastly, not interested in the study or in research. Five participants worked in management roles (i.e. clinic directors, managers), six held dual roles of practitioner and manager, and fourteen worked as practitioners. None of the participating clinics employed people whose exclusive position was 'researcher'. The average number of years working at their respective clinics was three. The level of research experience among participants varied, with seven of the participants reporting past research training. The type of research recognized by participants as important or relevant ranged from explorative descriptive type studies to randomized controlled trials. Five participants worked at two clinics that had an ongoing in-house outcomes research program and their degree of research literacy was higher. However, while these five people saw the value of research more clearly than the others, they shared similar insights as the other participants about the challenges of conducting research. Participants are indentified with a unique letter (A, B, C etc).

**Table 1:** Description of participating clinics

Location (Province)	Number of Practitioners	Modalities	Caseload	Who was interviewed
British Columbia	12	MD, ND, Chiro, Yoga, M/B, TCM, RMT	100-300	Physician Manager

British Columbia	3	MD, ND, TCM,	<100	CAM Practitioner

Alberta	3	M/B, Nutrition, ND	<100	CAM Practitioner
				CAM Practitioner- Manager

Alberta	11	ND, Nutrition, Dentist, TCM, M/B, MD	100-300	CAM Practitioner
				CAM Practitioner-Manager

Ontario	14	MD, RMT, TCM	100-300	CAM Practitioner

Ontario	10	Chiro, TCM, ND	100-300	CAM Practitioner-Manager

Ontario	9	ND, TCM, RMT, Chiro, MD, Yoga	>300	Manager
				Physician

Ontario	3	MD, M/B	<100	Physician

Ontario	8	ND, MD, RMT, TCM	100-300	CAM Practitioner-Manager

Ontario	3	MD, ND	<100	CAM- Practitioner

Ontario	11	MD, TCM, Phyio, M/B	100-300	CAM Practitioner-Manager

Ontario	3	MD, TCM, Physio, M/B	100-300	Manager-Clinic Director

Ontario	3	MD, ND, TCM	<100	CAM-Practitioner

Ontario	10	MD, RMT, TCM	>300	Clinic Director-Manager
				CAM Practitioner-Manager

Ontario	5	MD, ND, Physio, Chiro	>300	Physician

Ontario	7	MD, M/B, Physio, Chiro	>300	Physician-Manager

Quebec	2	MD, TCM, RMT, ND, Chiro, Physio	<100	CAM Practitioner

Quebec	19	RMT, TCM, ND	>300	Manager-Clinic Director

Nova Scotia	2	MD, ND, TCM	100-300	Physician

Glossary: MD (physician), ND (naturopath), Chiro (chiropractor), Physio (Physical Therapy), TCM (traditional Chinese medicine), RMT (registered massage therapist), M/B (mind/body).

### Perceived value and role of outcomes research

Discussion about the value and role of outcomes research in participants' clinics proved to be a controversial and emotionally charged topic. Practitioners raised questions regarding the perceived impact of conducting in-house research (or lack thereof) on clinical care and its benefits to the patient. They also mentioned the increased burden on patients and practitioners and the financial cost of doing research. However, despite these concerns, the majority of participants perceived in-house research as being critical to the future of IHC, highlighting it as integral to program improvement, patient outcomes and directly impacting the field of IHC itself:

*"I see outcomes research as the only way to really assess the success of our program. Sure we can see if our individual patients are happy and making progress but in order to get the full picture, we need to document the progress and the pitfalls in a formal way. I feel this is important not only to us as practitioners in this clinic but also as a way to bring more credence and I suppose evidence to this field. We have come a long way but I feel there is still so much more to be explored and understood." *(CAM Practitioner-Manager, Ontario-I)

Generally, participants supportive of in-house research had very clear ideas on what they would like to achieve through research and its resultant benefits. A few practitioners and administrators believed outcomes research could help them to understand the complex interrelationships and impact of the various healing modalities offered at their clinics, and would engage patients more meaningfully within the clinic and in their healing journey. They felt that research could also help them advocate for change in the health care system. Clinic administrators in particular, thought that research participation would enrich IHC staff's work, making it more interesting and relevant, resulting in enhanced job satisfaction and retention. They also felt that research could bring additional skills and new perspectives into their IHC setting.

On the other hand some practitioners did not see the need to conduct research at their clinic since they felt that observing improvements in the wellbeing of patients was proof enough:

*"The practice itself will succeed if the approach and treatment is successful. If we as a clinic cannot provide 'better' results than the traditional health care system, then we would not be able to charge for our services. The fact that we have established a thriving business and have 'success' stories, supersedes our need to collect research"*. (CAM Practitioner, Alberta-D)

An underlying sentiment throughout the interviews suggested that administrators felt that conducting research was the politically correct thing to do and expressed feeling some pressure when they compared themselves to their peers and to what other IHC clinics were doing. Participants who worked in management generally had a more favorable opinion about research than did the practitioners. Several practitioners commented that administrators seemed to *"look at research through rose colored glasses" *whereas they, as well as front line staff, shared a more realistic perspective about the practical implications of conducting research such as increased workload and lack of research skills. Despite the range of opinions about the value and role of research in the context of IHC clinics, the challenges of conducting research emerged as an important overarching theme in the interviews.

### Challenges to conducting outcomes research

Despite the range of opinions about the value and role of research in the context of IHC clinics, the challenges of conducting research emerged as an important theme in the interviews. These challenges were summarized under four emergent categories: organizational culture, organizational resources, organizational environment, and logistical issues.

### Organizational culture

Challenges stemming from the culture existing within an organization such as the philosophy of IHC and the value and belief system of the organization were common issues among participants.

### a) Philosophy of IHC

At an organizational level, many participants felt that the lack of research conducted at their clinic could largely be attributed to the philosophy or mandate of their clinic, which they observed did not value, recognize or support the need for an ongoing formative research process.

*"Research is not woven into the clinic's operating philosophy like patient education or outreach is. Since research has never been reflected in the clinic's overall governing mandate, the chances are slim that it will ever be reflected in any of our organizational activities. At this point, research does not fit with our organization." *(Manager, Ontario-J)

Therefore, research was not a priority nor was it embedded in the daily operations of the clinic.

*"Our focus is on healing patients. This is how we prioritize our time and energy. Research is secondary to providing results. We operate from a patient-based model and I don't really see where research can fit into that"*. (CAM Practitioner-Manager, Alberta-E)

Practitioners who were interested in outcomes research identified the clinic's culture as the main reason they had not incorporated research in their practice because *"we do not work in an environment that promotes and fosters a spirit of inquiry"*. (CAM Practitioner, Ontario-H)

Some participants felt that research was incompatible with the IHC philosophy, which emphasizes wellness and whole person healing, compared to research, which they viewed as reductionist and focused on isolated aspects of care and of the individuals rather than on the whole system:

*"There is a resistance to outcomes research at our clinic. Research doesn't mesh well with the integrative healing philosophy of whole person care. As clinicians, we evaluate patients as whole people, the sum of all parts, whereas outcomes research seems to want to break everything into individual parts. It doesn't fit with the philosophy of integration." *(CAM Practitioner-Manager, Ontario-L)

### b) Attitudes and beliefs

As indicated before, most practitioners observed how their own personal beliefs influenced their involvement in research. They saw themselves as clinicians or healers who valued and prioritized patient care, rather than researchers or academics who they perceived as being outcomes-based and results driven:

*"I am a healer. I studied healing systems not research. My reason for being here is to work with patients and help them along their healing journey that is our focus." *(Physician, British Columbia-A)

Several practitioners also feared that participating in a research study could be harmful to their patients' healing process and have a detrimental impact on the therapeutic relationship. At many of the clinics, patients were dealing with multiple health issues and as such, participants were particularly concerned about burdening patients unnecessarily with research forms and surveys. Many of these participants expressed their discomfort to encourage patients to participate in research since they were paying for clinical services:

*"A great deal of our patients are dealing with very complex health problems. It is a struggle for many to get through their days and their treatments. I feel that asking them to add this to their list of things to do would be unfair and unrealistic. We are trying to support them and create wellness for them, not to overburden them with paperwork and forms. And for what? How will they personally benefit from participation? It just doesn't seem to add up. I would rather have them focus on other things than research forms." *(CAM Practitioner, Quebec-S)

Practitioners tended to be more open to research if it did not interfere with the administration of and resources necessary for patient care:

*"If research can be obtained in a manner that provides minimal impact on the delivery of primary care to the patient, then it is clearly worthwhile. However, the moment that it interferes or removes resources from running the clinic and services to the patient, then its value is diminished." *(Physician, Ontario-Q)

Some participants (both managers and practitioners) found that study results introduced conflict within the clinic, as everyone had different ideas about the validity and interpretation of the findings. For some practitioners, research findings became a sore point if their specific area of expertise became a target. For example, data from one clinic's outcomes research revealed that patients were unhappy about receiving mixed messages and conflicting treatment protocols from the nutritionist, naturopath and acupuncturist. Several practitioners found that situations like this did not nurture support for the continuation of outcomes research due to fear of having their professional practice scrutinized. For some, this was enough of a hindrance to discourage them from pursuing or supporting further research projects.

### c) Relevance of conducting research

For those who were involved in research, the immediate usefulness and application of research findings to the clinic setting was elusive, resulting in disinterest in continued participation in research. Several participants realized at the end of their study that after collecting ongoing data from patients for an in-house research project, the findings did not have much relevance or applicability to their clinic and was unfortunately of little value to them in terms of understanding patient experiences and improving practice. In these instances, data were analyzed, results produced, but participants were left wondering,

*"We spend so much energy getting the data and eagerly await the analysis only to find we can't apply any of the results to our daily practice. The promise of useful findings to impact our practice was like the light at the end of the tunnel. What is the motivation then?" *(CAM Practitioner, Alberta-F)

### Organizational resources

When there was an interest in research, the lack of organizational resources to successfully conduct outcomes research was commonly identified as a key barrier.

### a) Funding

The majority of participating clinics were small in scale and operated within a business model that was dependent on revenue-generating services and activities. Consequently, they were limited by the minimal resources available to carry out additional activities such as establishing any type of outcomes research program. If there were extra funds in the budget, the clinic often had a long list of items that took priority over research:

*"The problem I see with research is mainly financial. How can we make money off of research? It costs us money because we would have to hire someone to carry it out for us and really, how do we get that money back? We are constantly trying to cut corners and save dollars every way we can, I just can't justify it right now with the current system. I have a long list of expenses that must be taken care of before I can even fathom introducing research into this clinic. Larger organizations which have more access to funding and more manpower are in a better position to take this on"*. (Clinic Director-Manager, Ontario-O)

### b) Compensation for services

One of the biggest issues for practitioners was related to the compensation structure at their clinics which did not consider conducting research or any other activities outside of direct patient care. Most of them were paid on a fee for service basis and had no paid time allocated for research activities. Furthermore, there was no formal or informal mechanism to reward or recognize this additional commitment and work. Therefore, research initiatives would have to take place outside of regular paid working hours without any compensation from the organization, which most participants did not favor.

*"We as a small independent clinic do not have the extra funds to hire someone to only conduct research which means ultimately, if we are to conduct research, the responsibility falls on the shoulders of the practitioners. However, I feel that integrative medicine, like many other health care fields, exists in a culture of productivity that I feel contradicts what research is all about. Research requires one to stop, reflect and discuss. In this field, productivity means taking care of our clients. That is rewarded, we aren't rewarded for sitting and thinking and documenting"*. (Clinic Director-Manager, Ontario-O)

### c) Research training and skills

Many of the participants expressed concerns that they were ill prepared to conduct or engage in research on their own as they had little or no previous research training or skills. There was also a lack of incentives within the organization to encourage the development or improvement of their research literacy and capacity skills required to develop relevant research studies or to apply findings to improve practice patterns or service delivery.

In clinics that pursued in-house research activities, lack of research training or access to experienced researchers significantly impeded the research process. For example, lack of research training led to discouragement after a significant amount of time had been committed to data collection, only to find out that the data were not useable:

*"We got to the end of the study and had collected all of this data on our patients but had no clue what to do with it..... We spoke with a couple of people from the university who pointed out some serious flaws in our design and data collection. We felt so discouraged by the end result that we gave up. Nobody wanted to pursue it. The data is still sitting in a box in our storage room. It was a disappointing experience for everyone involved. Such a waste." *(Physician, Ontario-R)

### d) Clinic's partnerships/linkages

A few participants felt that their lack of formal and informal linkages to academic institutions was a barrier to developing a sustainable research program. Several participants spoke of colleagues from other organizations that were partnered with researchers from local universities who helped them to write proposals and develop strong projects:

*"I see other clinics like ours who have partnered with the local university and they are now hooked into all sorts of research initiatives. That is the route to go. We don't have those connections and contacts and I really think that limits us in a huge way in terms of opportunity to get involved in research." *(CAM Practitioner-Manager, Ontario-M)

### e) Research materials

In addition to the issues of funding and staffing, participants cited having limited access to research related resources such as books, academic CAM journals, and electronic databases as another barrier to research activities.

### Organizational environment

Many participants felt that the environment within which their clinics operated was constantly shifting and evolving thus influencing their ability to participate in research. Two of the issues participants struggled with most were the nature of their patient population and high staff turnover.

### a) Nature of patient population

Clinics with previous research experience observed how the unpredictable nature of their patients' health concerns and irregularity of patient visits negatively impacted systematic follow-up of data collected. This reality made data collection and follow-up with patients challenging and was perceived as a possible threat to recruitment and retention of participants, ultimately impacting the quality of data:

*"Many of my patients are living with illness that is cyclical and unpredictable. Often times they come to see me when they most need help and then I may not hear from them for a year. This lack of consistency is a major hindrance to participating in research. I have trouble enough following up with them, I can't imagine what it would be like to chase after them to get a questionnaire filled out!" *(CAM Practitioner, British Columbia-C)

### b) High staff turnover

Several participants also felt that the high rate of staff turnover could pose a threat to the quality of research collected. The rate of turnover was high at many of the participating clinics and participants were concerned that being short staffed and frequently training new employees would negatively impact the flow and integrity of research:

*"We work in an unpredictable, unstable environment. The loss of one staff person creates a hiccup in the research system and will inevitably impact results as we are often spread thin and the first thing to fall by the wayside is the research. Then we start up again once someone is trained and the cycle repeats itself." *(Manager-Clinic Director, Ontario-N)

### Logistical challenges

A significant stumbling block to conducting research for participants with previous research experience was integrating research protocols into the clinic's established routine. This was especially a challenge for the front office support staff and clinicians, as their daily activities were impacted more directly. Despite the administration's strong interest in and support for research, research did not seem to fit into the daily practice and activities for many of the participants. For many, superimposing a research program onto an existing IHC program was unrealistic and unsustainable. Participants anticipated major complications as a result of introducing extra steps for research into their "well oiled machine":

*"At the clinic, everyone has their own tasks to take care of. It is finally running smoothly. Why would I want to introduce an extra layer to complicate things? It is not a priority largely for this reason. Who has the time to take this on? We struggle so much just to stay afloat at this clinic. Why kill ourselves even more with adding on more?" *(Manager-Clinic Director, Quebec-T)

### Strategies to support outcomes research

Although participants identified a significant number of barriers to creating a research culture, they also provided insights into ways in which clinics could encourage the development of research culture.

### a) Develop and adopt a research philosophy

If clinics were to have any success in future research endeavors, they must first adopt and embody a research philosophy that emphasizes the value of research and integrates it into the organization's mission.

*"Valuing research as an intrinsic component of the organization is critical because values and philosophy are what guide the staff, practitioners and patients within the clinic and gets us thinking research. It should be part and parcel of what we do as an organization. No questions asked. Clinical care and research are intimately linked and they feed each other. Staff, time and infrastructure for research related activities needs to be factored into the budget." *(Manager, British Columbia-B)

### b) Organizational leaders as research advocates

It is crucial that a belief in a culture of research be reflected in the clinic's leadership. Clinic administrators need to become research advocates, creating a supportive research environment, ensuring resources (including personnel, time, financial and administrative support) are allocated and effectively implemented into the daily practice of the administrators, practitioners and front office support staff.

*"It is the leader's job to make sure that research receives equal attention and importance within the clinic as all other aspects including patient education and patient care"*. (CAM Practitioner-Manager, Ontario-P)

Participants suggested that having dedicated staff to administer, manage and seek research opportunities could facilitate productivity and program sustainability:

*"Appointing a person whose main role is to spearhead the research process would help our clinic immensely. The person could look for grant opportunities and linkages and get the ball rolling for us." *(CAM Practitioner-Manager, Alberta-G)

### c) Develop viable research processes and structures

In order for research findings to be meaningful and have a direct impact on practice, the research process must be collaborative, consensus-driven and inclusive, where individuals are encouraged to contribute to the research program. Also, it is essential to identify the needs and desired outcomes of the clinic prior to designing the research such that it reflects actual practice but is also responsive to the questions that the clinic team considers important and relevant. Participants felt that such an approach would encourage individuals to become more invested in the research process.

Research activities need to be aligned with clinical workflow processes at an early stage in order to minimize the potentially negative impact of adding new tasks to individuals' workload, particularly if they are not compensated. Research activities should be embedded into administrative and practice duties of front office support staff and practitioners, and explicitly outlined in the clinic policy and procedure manuals. Research protocols should be streamlined with clinical protocols. For example, consent for clinical purposes and research purposes should be merged into one form. In order to reduce the administrative burden on patients, ideally, outcomes collected for research purposes should be useful to practitioners as well. In this way, the research data are directly applicable to practice.

Furthermore as one participant emphasized, clear protocols on how decisions are made about research, how projects will be supported, and how their findings will be handled, are paramount:

*"This kind of formality and process will show a commitment to staff that the results of the research will weigh heavily in organizational decision making and where action will be taken accordingly. We also will be clear on decisions made about handling the final results." *(Physician, British Columbia-A)

Research within an IHC clinic should be encouraged by positive reinforcement and recognition. Several participants suggested that reward systems be introduced to encourage initiative and ongoing participation. They also identified positive accolades from colleagues to be important reinforcement and encouragement. Interest, praise and recognition were highly valued rewards:

*"I think more than anything, I would just like to be acknowledged for my extra work. I did a workshop for my team here based on some things that I learned at a research conference I attended and did I ever receive positive feedback. That type of recognition felt good and has encouraged me to attend more research related events." *(Physician, Ontario-K)

Creating access to research resources was critical. Having access to academic CAM journals, establishing productive relationships with academic "mentors"/researchers, and access to research funds were key resources identified by most participants. Because funding and sufficient staffing were major issues for the participating clinics, several participants suggested the creation of large 'umbrella' research projects based in academic institutions in which smaller, individual IHC clinics could take part. In addition, participants discussed the possibility of having access to previously funded grant applications to use as a template to guide their own in-house proposal development.

## Discussion

Balancing the development of clinic services and research activities remains a challenge for most IHC clinics. Securing resources to do both at the same time is challenging, and superimposing research activities on clinic work processes without adding resources such as staff, space, expertise and time can be a stressful experience for clinicians and administrative staff. Identifying and actively removing the barriers to conducting outcomes research in IHC settings will be key to generating new knowledge in the field.

Many studies evaluating IHC were conducted by people outside of IHC clinics, often by students [22,24]. This raises an interesting question about research initiated in-house versus research conducted by outsiders: Does the latter address or identify the needs of the clinic equally well? Our team consisted of a mixture of these, which was a major advantage in reflecting upon the ways in which our own values, experiences, interests, beliefs and social identities could have shaped our research. One of us (BF) is a nurse and has directed an Integrative Clinic in the early 2000s. SM is a CAM practitioner and researcher and is involved in developing, implementing and assessing different models of IHC in primary care community settings. AM and MV are not practitioners and have conducted research in IHC for the past 8 years. AK has worked as a CAM practitioner in an integrated health care setting. We believe that the range of our roles and our close collaboration has made it possible for us to maintain a critical and thoughtful attitude towards data collection and analysis.

Our earlier 'optimal outcomes' study set out to address previously identified barriers by choosing measures and research instruments that would more closely capture the patients' lived experiences, however, despite all these considerations, we still encountered significant barriers in implementing the study protocol within IHC sites. This study served to identify the subtle, yet complex issues that continue to hinder evaluation of IHC service delivery models. The relatively low response rate in this study further suggests the lack of interest in or relevance of research in the IHC context. Identifying these barriers proved to be very informative and may contribute to a more complete understanding of what "*readiness*" to conduct research practically looks and feels like in an IHC clinic [25].

We found that almost all barriers to conducting outcomes research are directly or indirectly correlated with the IHC clinic's organizational culture as it relates to evaluation and/or research. These include the research-related beliefs and values of staff, the degree of research literacy amongst the practitioners and individuals in management roles, and experience using quality management strategies to improve programs and services. Some would argue that the underpinnings of a supportive research culture should be introduced during a health professional's basic education and that practitioners' education should begin to develop research literacy and capacity. Many CAM institutions focus on training students to practice rather than conducting research, thus they have had little exposure to the relevance and need for research in this context during their training [26]. In education programs for conventional health care providers/practitioners (such as physicians and nurses), research is often presented as an information source to be used in practice but produced "outside" of the practice setting and by "others". It is rarely taught as a component of the clinical practice for the purpose of internal evaluation or to contribute to a broader knowledge base within a given field.

As this study highlights, it is critical for IHC clinics to adopt research as a priority at the leadership level and to build in research capacity from the inception of the clinic. Capacity building activities could include formal mentorship by resident scholars; opportunities for all staff to serve as members of the research team to help design protocols and analyze results; journal clubs that introduce relevant literature and point out gaps in the literature; and, lastly, creating linkages to existing research programs or centers. Translating results into action is key to the sustainability of research. As illustrated by the data in this study, providing patients with a platform to provide ongoing feedback and share their healing experiences, clinics are creating endless opportunities to improve practice. Leaders that adequately weave research goals and related activities through the fabric of the organization will be more successful in creating an evaluation culture, which will in turn inform on-going development of effective services and programs.

The literature on IHC provides little practical guidance to IHC clinics about how to incorporate a viable research component within an IHC program. However, the fields of primary care, social work, higher education, and kinesiology have explored factors needed to build research capacity and develop a research culture in practice settings [17-21]. Barriers to research in these fields have been identified, such as lack of time to conduct research, staffing resources, and lack of interest among staff. It has been determined that protected, dedicated research time, effective managerial support, research training, immediate access to mentorship and a nurturing workplace environment are key to a sustainable research culture [17-21]. These findings draw numerous parallels to those identified in this study, demonstrating the need for individuals working in IHC to seek out knowledge and experience from those in other fields. Based on the findings of this study and those in other fields, the next step in fostering the development of research cultures in IHC clinics could be to develop a set of practical guidelines for clinic managers and directors outlining feasible approaches and options of how to implement a research arm within their clinic setting.

## Conclusions

There is increasing public and professional demand to identify the most effective ways to deliver IHC, and agreement that evaluating the outcomes of IHC clinics is a critical step towards creating this new knowledge. In an ideal world, established IHC clinics would respond to this call for evidence by agreeing upon a set of relevant, easy to administer evaluation tools, and collaborate on collecting much-needed outcomes data. However, in an emerging field of study such as IHC with a diverse and broad range of options, this may not be possible in the short term. We acknowledge, from our own lived experiences as supported by these study findings, that IHC clinics are highly complex systems. Designing studies that take into consideration and respond to the multitude of factors and components that contribute and create an IHC system is needed to develop an in-depth and comprehensive understanding of IHC and the potential patient outcomes. Research approaches that view IHC from a whole systems perspective [27] are more likely to be experienced by stakeholders as 'respectful processes' and in turn, produce more useful knowledge.

As highlighted by this study, clinical and research pioneers of IHC are recognizing that creating a viable evaluation culture in tandem with developing clinic services is an important pre-cursor to successfully conducting outcomes research in complex IHC settings.

## Competing interests

The authors declare that they have no competing interests.

## Authors' contributions

MJV conceptualized and designed the study, participated in data analysis, and drafted the manuscript. AM assisted in the study design and coordination, collected and analyzed data, and drafted the manuscript. AK coordinated project activities, collected data, and helped draft the manuscript. BF was involved in the conceptualization of the study and manuscript review. SM was involved in the original study and reviewed the manuscript. All authors have read and approved the final manuscript submitted.

## Pre-publication history

The pre-publication history for this paper can be accessed here:

http://www.biomedcentral.com/1472-6963/10/14/prepub

## Supplementary Material

Additional File 1 **List of outcome measures included in the "Outcomes Package"**. A comprehensive list of the outcome measures and a brief description of each that was included in the Outcomes Package which was used in the initial pilot study [8,28-34]. Click here for file

Additional File 2 **Interview Guide**. The final questions which were developed and used as a guide for the semi-structured telephone interviews with all participants. Click here for file
