# Supplementary material for: Identifying the barriers to conducting outcomes research in integrative health care clinic settings - a qualitative study
Source: BMC Health Serv Res. 2010 Jan 14;10:14. doi: 10.1186/1472-6963-10-14 (PMC2826302; doi:10.1186/1472-6963-10-14)
Supplement: Additional File 1 — List of outcome measures included in the "Outcomes Package". A comprehensive list of the outcome measures and a brief description of each that was included in the Outcomes Package which was used in the initial pilot study [8,28-34]. [file 1472-6963-10-14-S1.DOC]

## Additional File 1 –

## Figure 1 - List of outcome measures included in the “Package of Outcome Measures”

| **Outcome Measure** | **Description** |
| --- | --- |
| SF-12 [28] | Designed as a generic indicator of health status measuring eight dimensions of quality of life, it is a reputable measure and widely used in conventional medicine. |
| Arizona Integrative Outcomes Scale [29] | Assesses self-rated global sense of wellbeing |
| Numeric Pain Distress Scale | Rates pain intensity on a numbered scale |
| Energy Visual Analogue | Self designed at Tzu Chi Institute |
| Social Support Question | Self designed at Tzu Chi Institute |
| Stress Visual Analogue | Self designed at Tzu Chi Institute |
| Spitzer’s Quality of Life UniScale [30] | Global overall measure of quality of life |
| Control Preferences Scale [31] | Adapted from a scale that measures the degree of control an individual, who is living with a life threatening illness, wants to assume when decisions are being made about medical treatment. |
| Patient Enablement Inventory [32] | An outcome measure that evaluates degree patients feel enabled by experience with practitioner |
| Adapted measure based on the Picker Patient Satisfaction Survey [33] | Adapted from the Picker Institute’s Patient Satisfaction Scale |
| Measure Your Own Medical Outcomes Profile (MYMOP) [8] | A tool that aims to measure the outcomes that the patient identifies the most important |
| Measure Your Own  Transformational Outcome Profile [34] | A tool that aims to measure any significant changes a patient has identified as experiencing while receiving care |
