# Supplementary material for: Identifying the barriers to conducting outcomes research in integrative health care clinic settings - a qualitative study
Source: BMC Health Serv Res. 2010 Jan 14;10:14. doi: 10.1186/1472-6963-10-14 (PMC2826302; doi:10.1186/1472-6963-10-14)
Supplement: Additional File 2 — Interview Guide. The final questions which were developed and used as a guide for the semi-structured telephone interviews with all participants. [file 1472-6963-10-14-S2.DOC]

## Additional File 2 –

## Figure 3 - Interview Guide

| 1. Can you please start by giving me some background information on the clinic and the programs offered here? (Population, size, how long in operation, health care team) 2. What is the organization’s mission and philosophy toward the work that you do here? 3. How would you describe the model of care delivery that your clinic follows? For example: is it self-standing or are you aligned with one of the hospitals or universities? 4. How does the team operate (at intake and during the treatment process?) – as a team, as separate individuals? 5. What is the communication model for practitioners at the clinic? For example: do you have weekly meetings to discuss cases or only meet informally in the hallways? 6. What would you say are the most important patient centered outcomes? 7. How would you describe your understanding of research? 8. Do you conduct research at your clinic? If yes, what kind of research? Outcomes research? If not, why not? If you were to develop a research program, what type of research do you see working with your model? 9. Do you feel that your clinic has a culture that supports research and inquiry? Why do you feel this way? 10. What do you feel has been done to facilitate such a culture? (what supports exist?) 11. What barriers, if any, have you encountered when creating a research culture/ can you foresee hindering the research process? Prompts: "no incentives for patients, no incentives for practitioners, time involved for patients and practitioners, so many vast areas to research in the field- disagreement among staff as to what route to go". 12. How do you involve patients and practitioners in research? How do you foresee involving patients and practitioners in research? 13. In a perfect world. What kind of supports would you need in place or available to you to create a health, sustainable research culture? |
| --- |
